# Supplementary material for: Plasmids in the human gut reveal neutral dispersal and recombination that is overpowered by inflammatory diseases
Source: Nat Commun. 2024 Apr 11;15:3147. doi: 10.1038/s41467-024-47272-x (PMC11009399; doi:10.1038/s41467-024-47272-x)
Supplement: Supplementary file 1 — Supplementary Information [file 41467_2024_47272_MOESM1_ESM.pdf]

Supplementary Information

Plasmids in the human gut reveal neutral dispersal and recombination that is overpowered by inflammatory diseases

Zorea et al.

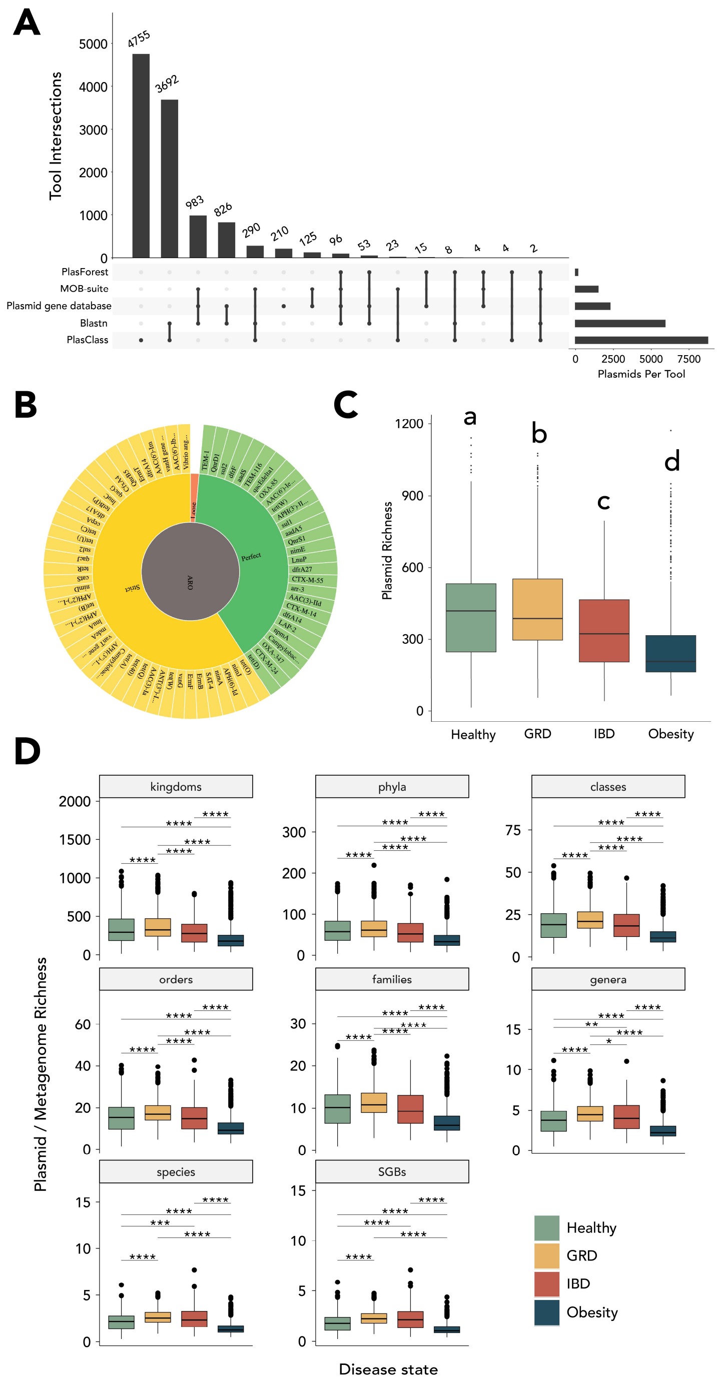

Supplementary Figure 1. Plasmids assembled in this study and the functions they encode. (A) The overlap of plasmids predicted by SCAPP and other plasmid tools. The connected horizontal bars represent intersections between the tools, and the numbers above the bars indicate the count of plasmids found in the corresponding intersection. The vertical bar on the leftmost side represents the total count of plasmids identified collectively by all tools. (B) Output of the Resistance Gene Identifier (RGI) depicting the antibiotics found on plasmids in this study, as well as the detection paradigm (perfect / strict / loose). (C) Plasmid richness distributions within each disease state (two-sided Wilcoxon rank-sum test, false discovery rate (FDR) corrected $p < 0.001$). Midlines of boxplots represent the median, boxes the interquartile range (25th to 75th percentile), and whiskers the range of data. n = 1,548 healthy, 339 IBD (Inflammatory Bowel Disease), 1,035 GRD (Glucose-metabolism Related Diseases), and 545 obese individuals. (D) Plasmid/species richness distributions within each disease state, for different phylogenetic levels (two-sided Wilcoxon rank-sum test, FDR corrected, $p < 0.05$ (*), $p < 0.01$ (**), $p < 0.001$ (***) or $p < 0.0001$ (****)). Midlines of boxplots represent the median, boxes the interquartile range (25th to 75th percentile), and whiskers the range of data. n = 1,548 healthy, 339 IBD, 1,035 GRD, and 545 obese individuals.

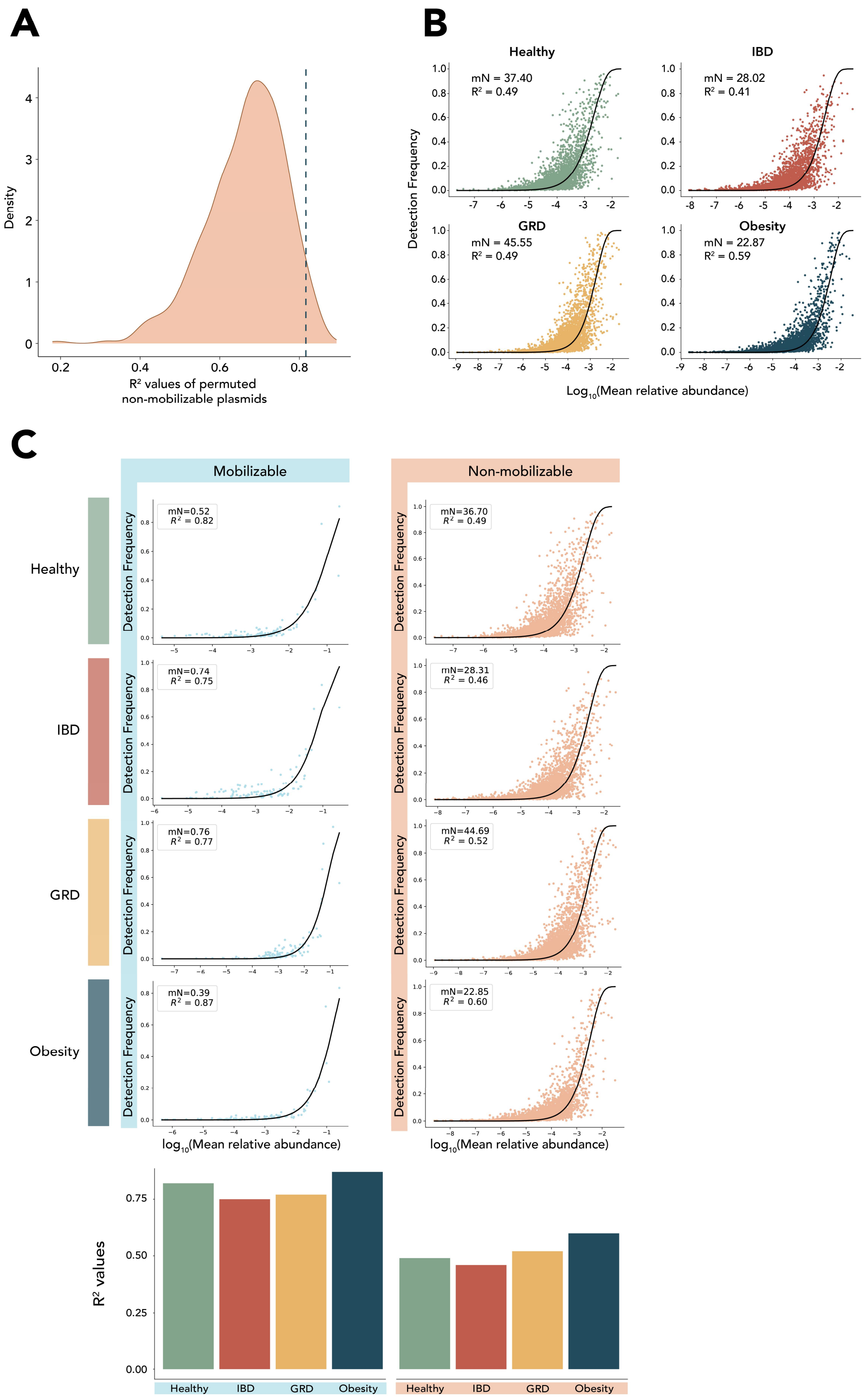

Supplementary Figure 2. NCM fits as a function of disease state and plasmid lifestyle. (A) R^2 values of the neutral community model (NCM) fits of 123 non-mobilizable plasmids, randomly selected in 1,000 iterations. The dashed line represents the R^2 value of the mobilizable plasmids. (B+C) NCM fit of plasmids, computed on individuals of each disease state (B), as well as separately for each plasmid lifestyle (C). Each dot represents a plasmid, and the solid lines indicate the best fit to the NCM. R^2 values measure the goodness of fit to the neutral model and the mN values are the migration rates from global to local patches (metacommunity size times immigration). The barplot depicts these R^2 values as a function of disease state and plasmid lifestyle. Abbreviations: IBD, Inflammatory Bowel Disease; GRD, Glucose-metabolism Related Diseases.

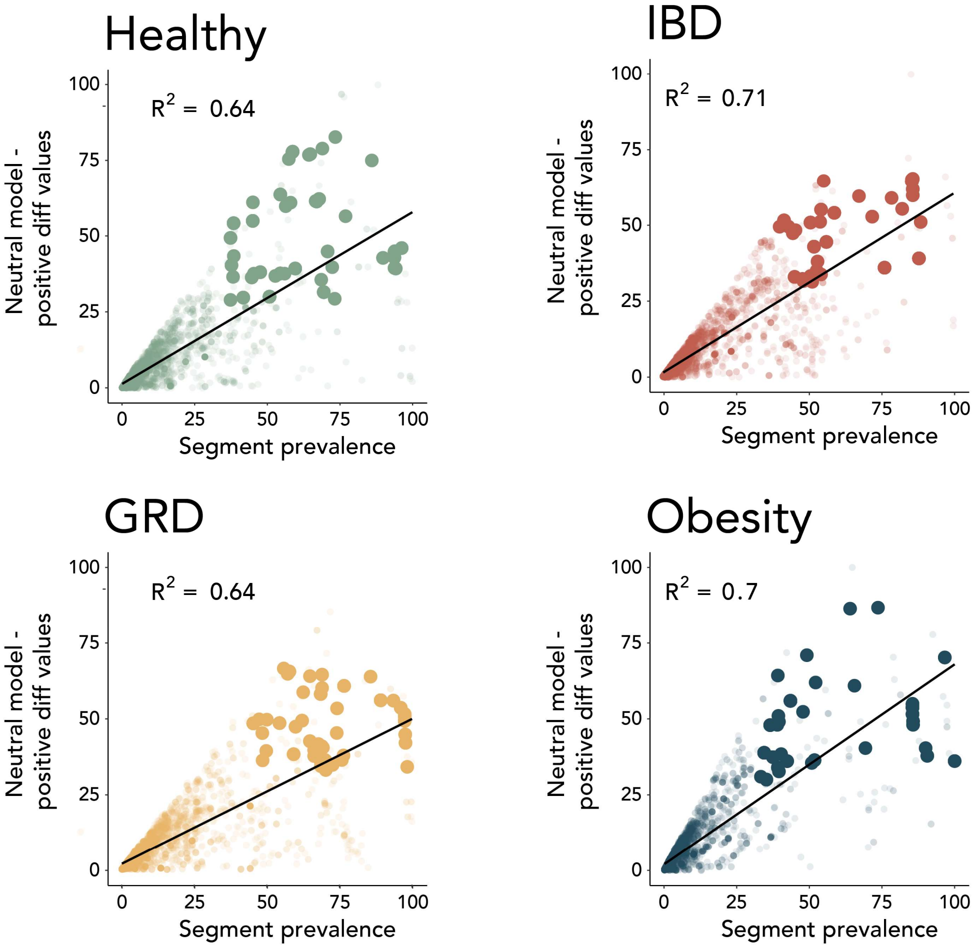

Supplementary Figure 3. Segments that deviate from neutrality as predicted by NCM fits are also prevalent across individuals. The linear correlations between segment prevalence across individuals within each disease state, and their deviations from the neutral community model (NCM) fit, with only positive deviations shown (determined by their distance from the fit on the y-axis). Each dot represents a segment, separated by disease state. The larger dots indicate segments that have been identified as being under selection, based on their placement in the top 5% for both prevalence and deviation values from the neutral fit. Abbreviations: IBD, Inflammatory Bowel Disease; GRD, Glucose-metabolism Related Diseases.

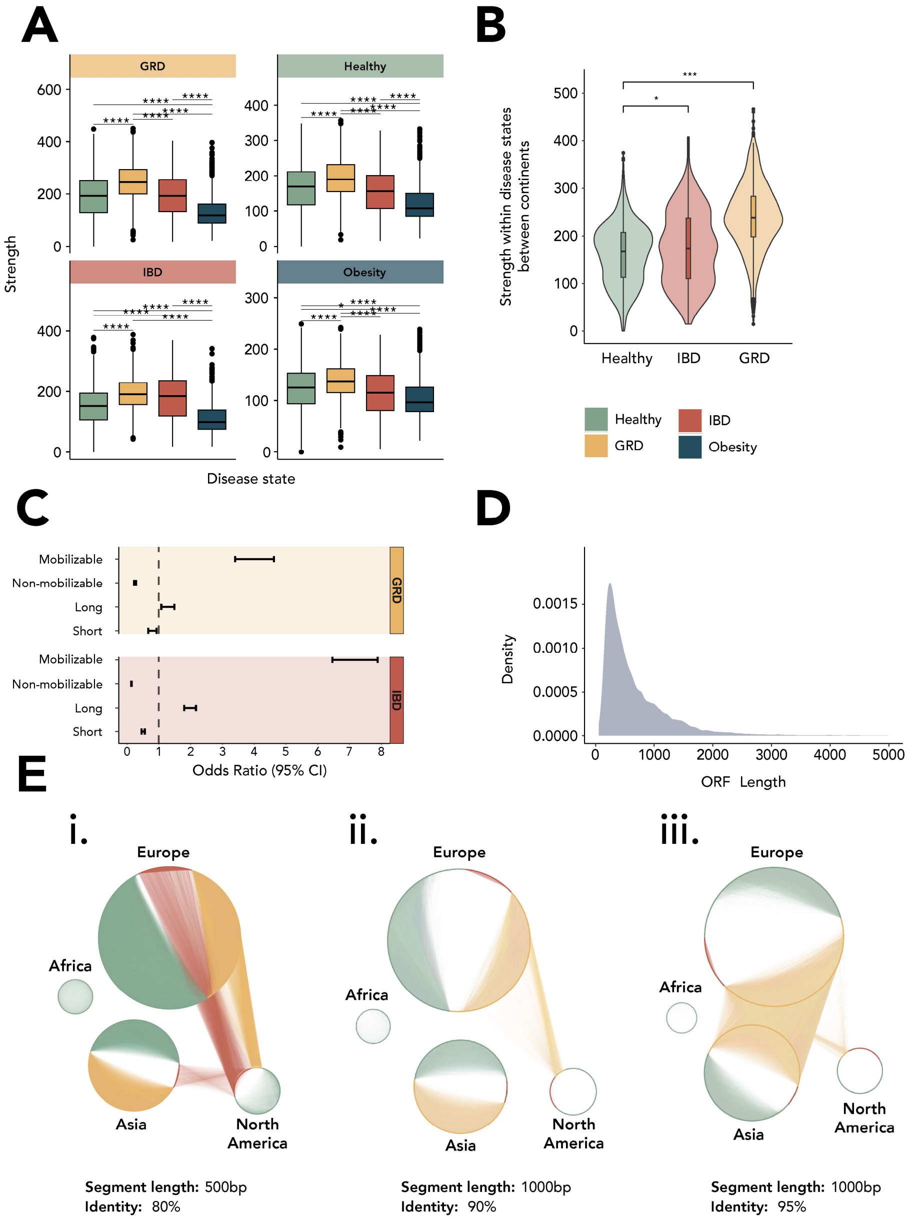

Supplementary Figure 4. Connectivity of individuals within the similarity network as a function of disease state and continent. (A) The strengths (sum of edge weights per each node) of nodes in the similarity network as a function of disease state (two-sided Wilcoxon rank-sum test, false discovery rate (FDR) corrected, p < 0.05 (*), p < 0.01 (**), p < 0.001 (***) or p < 0.0001 (****)). Midlines of boxplots represent the median, boxes the interquartile range (25th to 75th percentile), and whiskers the range of data. The charts are divided based on the disease state of the examined node, and the boxplots are color-coded to represent the disease state of the connecting nodes. n = 1,548 healthy, 339 IBD, 1,035 GRD, and 545 obese individuals. Abbreviations: IBD, Inflammatory Bowel Disease; GRD, Glucose-metabolism Related Diseases. (B) A violin plot showing the strengths between individuals of the same disease state and different continents (two-sided Wilcoxon rank-sum test, FDR corrected, p < 0.05 (*) or p < 0.001 (***)). Boxplots indicate the median and quartiles, with whiskers reaching up to 1.5 times the interquartile range. The violin plot outlines illustrate kernel probability density, i.e. the width of the shaded area represents the proportion of the data located there. n = 1,548 healthy, 339 IBD, 1,035 GRD, and 545 obese individuals. (C) Odds ratios of plasmid lengths (long plasmids >10kbp) and lifestyles of significant cross-continental segments in the diseases IBD and GRD against the overall distribution of plasmid lengths and lifestyles in the dataset. (D) The distribution of plasmid ORF lengths. (E) Similarity networks of shared plasmid segments of i) 500bp in size, 80% identity, ii) 1000bp in size, 90% identity and iii) 1000bp in size, 95% identity. Nodes represent humans and the edges that connect between humans of the same disease state encode shared plasmid segments. The weights of these edges represent the number of shared segments between any two individuals.

For visualization purposes only, edges that appear in this network connect between humans if they share at least 100 segments (weight>100) and are part of a group of disease states and continents that exhibit significant connectivity compared to a null model ($p < 0.0001$, FDR corrected). Nodes are grouped by their continent of origin and are colored according to their disease states.

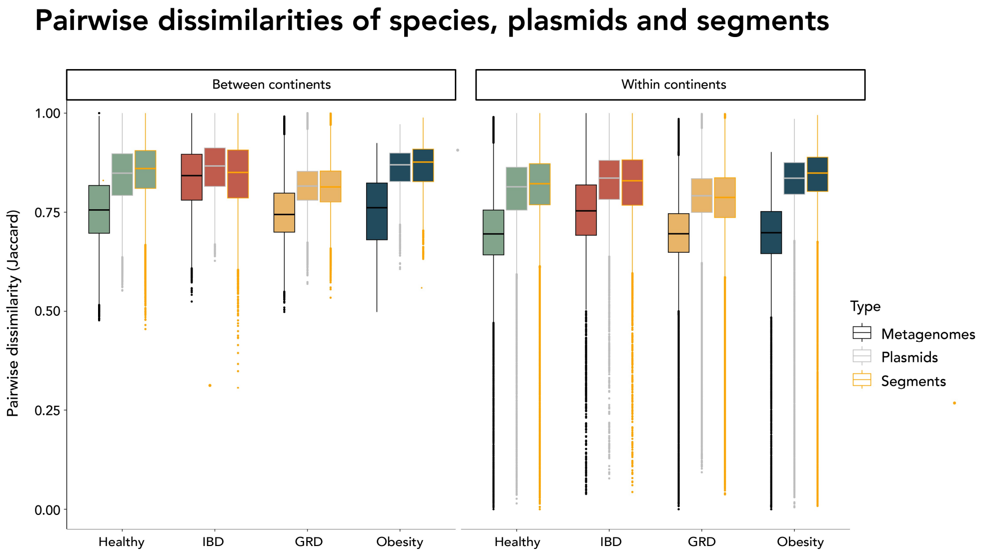

Supplementary Figure 5. Examining dissimilarities between distinct genetic entities reveals partial independent dispersal of plasmid segments from their microbial hosts. Pairwise dissimilarity scores (Jaccard index) between individuals of different disease states that originate from different (left) and the same (right) continent, calculated separately for metagenomes (black), plasmids (grey), and segments (yellow). Abbreviations: IBD, Inflammatory Bowel Disease; GRD, Glucose-metabolism Related Diseases.

Project ID	Samples	Reference
PRJEB10878	53	Yu et al. 2017[1]
PRJEB12124	172	Gu et al. 2017[2]
PRJEB1220	688	Nielsen et al. 2014[3]
PRJEB12947	33	Palleja et al. 2016[4]
PRJEB15371	87	He et al. 2017[5]
PRJEB17784	69	Bedarf et al. 2017[6]
PRJEB1786	135	Karlsson et al. 2013[7]
PRJEB18755	18	Kuang et al. 2017[8]
PRJEB2054	124	Qin et al. 2010[9]
PRJEB4336	387	Le Chatelier et al. 2013[10]
PRJEB7774	38	Feng et al. 2015[11]
PRJEB7949	40	Unpublished data
PRJNA196801	26	Minot et al. 2013[12]
PRJNA278393	30	Rampelli et al. 2015[13]
PRJNA290729	15	Louis et al. 2016[14]
PRJNA299502	23	Sankaranarayanan et al. 2015[15]
PRJNA305507	41	Petersen et al. 2017[16]
PRJNA319574	284	Schirmer et al. 2016[17]
PRJNA321058	52	Vaughn et al. 2016[18]
PRJNA324129	62	Maldonado-Gómez et al. 2016[19]
PRJNA328899	48	Liu et al. 2016[20]
PRJNA339012	4	Kumar et al. 2017[21]
PRJNA356102	8	Han et al. 2022[22]
PRJNA361402	575	Wu et al. 2017[23]
PRJNA422434	366	Qin et al. 2012[24]
PRJNA690543	89	Jacobson et al. 2021[25]

Supplementary Table 1. Publicly available gut metagenomic datasets that were used in this study.

**All samples**	**3,467**
**Continents**	
Europe	2,397
Asia	752
Africa	110
North America	208
**Disease states**	
Healthy	1,548
Inflammatory Bowel Disease:	
Crohn's disease	151
Ulcerative colitis	168
Unspecified	20
Glucose-metabolism Related Diseases:	
Impaired glucose tolerance	46
Type II diabetes	989
Obesity	545
**Gender**	
Male	1,247
Female	1,228
Unspecified	992

Supplementary Table 2. Association of continent, disease state, and gender of individuals in this study.

Supplementary References

1. Yu, J. *et al.* Metagenomic analysis of faecal microbiome as a tool towards targeted non-invasive biomarkers for colorectal cancer. *Gut* **66**, 70–78 (2017).
2. Gu, Y. *et al.* Analyses of gut microbiota and plasma bile acids enable stratification of patients for antidiabetic treatment. *Nat. Commun.* **8**, 1785 (2017).
3. Nielsen, H. B. *et al.* Identification and assembly of genomes and genetic elements in complex metagenomic samples without using reference genomes. *Nat. Biotechnol.* **32**, 822–828 (2014).
4. Palleja, A. *et al.* Roux-en-Y gastric bypass surgery of morbidly obese patients induces swift and persistent changes of the individual gut microbiota. *Genome Med.* **8**, 67 (2016).
5. He, Q. *et al.* Two distinct metacommunities characterize the gut microbiota in Crohn's disease patients. *Gigascience* **6**, 1–11 (2017).
6. Bedarf, J. R. *et al.* Functional implications of microbial and viral gut metagenome changes in early stage L-DOPA-naïve Parkinson's disease patients. *Genome Med.* **9**, 39 (2017).
7. Karlsson, F. H. *et al.* Gut metagenome in European women with normal, impaired and diabetic glucose control. *Nature* **498**, 99–103 (2013).
8. Kuang, Y.-S. *et al.* Connections between the human gut microbiome and gestational diabetes mellitus. *Gigascience* **6**, 1–12 (2017).
9. Qin, J. *et al.* A human gut microbial gene catalogue established by metagenomic sequencing. *Nature* **464**, 59–65 (2010).
10. Le Chatelier, E. *et al.* Richness of human gut microbiome correlates with metabolic markers. *Nature* **500**, 541–546 (2013).
11. Feng, Q. *et al.* Gut microbiome development along the colorectal adenoma–carcinoma sequence. *Nat. Commun.* **6**, 1–13 (2015).
12. Minot, S. *et al.* Rapid evolution of the human gut virome. *Proc. Natl. Acad. Sci. U. S. A.* **110**, 12450–12455 (2013).
13. Rampelli, S. *et al.* Metagenome Sequencing of the Hadza Hunter-Gatherer Gut Microbiota. *Curr. Biol.* **25**, 1682–1693 (2015).
14. Louis, S., Tappu, R.-M., Damms-Machado, A., Huson, D. H. & Bischoff, S. C. Characterization of the Gut Microbial Community of Obese Patients Following a Weight-Loss Intervention Using Whole Metagenome Shotgun Sequencing. *PLoS One* **11**, e0149564 (2016).
15. Sankaranarayanan, K. *et al.* Gut Microbiome Diversity among Cheyenne and Arapaho Individuals from Western Oklahoma. *Curr. Biol.* **25**, 3161–3169 (2015).
16. Petersen, L. M. *et al.* Community characteristics of the gut microbiomes of competitive cyclists. *Microbiome* **5**, 98 (2017).

17. Schirmer, M. *et al.* Linking the Human Gut Microbiome to Inflammatory Cytokine Production Capacity. *Cell* **167**, 1897 (2016).
18. Vaughn, B. P. *et al.* Increased Intestinal Microbial Diversity Following Fecal Microbiota Transplant for Active Crohn's Disease. *Inflamm. Bowel Dis.* **22**, 2182–2190 (2016).
19. Maldonado-Gómez, M. X. *et al.* Stable Engraftment of Bifidobacterium longum AH1206 in the Human Gut Depends on Individualized Features of the Resident Microbiome. *Cell Host Microbe* **20**, 515–526 (2016).
20. Liu, W. *et al.* Unique Features of Ethnic Mongolian Gut Microbiome revealed by metagenomic analysis. *Sci. Rep.* **6**, 34826 (2016).
21. Kumar, R. *et al.* Identification of donor microbe species that colonize and persist long term in the recipient after fecal transplant for recurrent Clostridium difficile. *NPJ Biofilms Microbiomes* **3**, 12 (2017).
22. Han, M. *et al.* The Potential of Gut Microbiota Metabolic Capability to Detect Drug Response in Rheumatoid Arthritis Patients. *Front. Microbiol.* **13**, 839015 (2022).
23. Wu, H. *et al.* Metformin alters the gut microbiome of individuals with treatment-naive type 2 diabetes, contributing to the therapeutic effects of the drug. *Nat. Med.* **23**, 850–858 (2017).
24. Qin, J. *et al.* A metagenome-wide association study of gut microbiota in type 2 diabetes. *Nature* **490**, 55–60 (2012).
25. Jacobson, D. K. *et al.* Analysis of global human gut metagenomes shows that metabolic resilience potential for short-chain fatty acid production is strongly influenced by lifestyle. *Sci. Rep.* **11**, 1724 (2021).
