# Supplementary material for: Plasmids in the human gut reveal neutral dispersal and recombination that is overpowered by inflammatory diseases
Source: Nat Commun. 2024 Apr 11;15:3147. doi: 10.1038/s41467-024-47272-x (PMC11009399; doi:10.1038/s41467-024-47272-x)
Supplement: Supplementary file 3 — Reporting Summary [file 41467_2024_47272_MOESM3_ESM.pdf]

nature portfolio

Corresponding author(s): Itzik Mizrahi

Last updated by author(s): Mar 3, 2024

Reporting Summary

Nature Portfolio wishes to improve the reproducibility of the work that we publish. This form provides structure for consistency and transparency in reporting. For further information on Nature Portfolio policies, see our Editorial Policies and the Editorial Policy Checklist.

Statistics

For all statistical analyses, confirm that the following items are present in the figure legend, table legend, main text, or Methods section.

n/a	Confirmed	
☐	☒	The exact sample size (*n*) for each experimental group/condition, given as a discrete number and unit of measurement
☐	☒	A statement on whether measurements were taken from distinct samples or whether the same sample was measured repeatedly
☐	☒	The statistical test(s) used AND whether they are one- or two-sided<br>*Only common tests should be described solely by name; describe more complex techniques in the Methods section.*
☒	☐	A description of all covariates tested
☐	☒	A description of any assumptions or corrections, such as tests of normality and adjustment for multiple comparisons
☐	☒	A full description of the statistical parameters including central tendency (e.g. means) or other basic estimates (e.g. regression coefficient) AND variation (e.g. standard deviation) or associated estimates of uncertainty (e.g. confidence intervals)
☐	☒	For null hypothesis testing, the test statistic (e.g. *F*, *t*, *r*) with confidence intervals, effect sizes, degrees of freedom and *P* value noted<br>*Give P values as exact values whenever suitable.*
☒	☐	For Bayesian analysis, information on the choice of priors and Markov chain Monte Carlo settings
☒	☐	For hierarchical and complex designs, identification of the appropriate level for tests and full reporting of outcomes
☐	☒	Estimates of effect sizes (e.g. Cohen's *d*, Pearson's *r*), indicating how they were calculated

Our web collection on statistics for biologists contains articles on many of the points above.

Software and code

Policy information about availability of computer code

Data collection	No software was used for data collection.
Data analysis	Paired-end reads were trimmed and cleaned using Trim Galore v2.6 and assembled into contigs by Megahit v1.0.3. Plasmids were assembled by SCAPP v0.1.4. These plasmids were annotated or assembled using additional programs: MOB-suite, Blastn, PlasForest, and PlasClass. The overlap between different plasmid assemblers was plotted using the "UpSetR" R package. Plasmids were deduplicated using BLASTn v2.10.1+, and reads were mapped to them using BBmap v38.86. Their abundance per sample was determined using Metabat2 v2.12.1. The read coverage of plasmids in each sample was computed by SAMtools mpileup v1.10.<br>We utilized MOB-suite v3.0.3 to classify plasmids as mobilizable or non-mobilizable. Open Reading Frames were predicted by Prokka v1.12. Annotations were achieved using anvi'o v7.1. These steps were all run in parallel using the NeatSeq-Flow workflow platform. AMR genes were predicted using the Resistance Gene Identifier (RGI) v6.0.1. Read taxonomies were determined by MetaPhlAn v4.0.3. Segments were determined by BLASTn results and clustered using cd-hit-est v4.8.1.<br>Statistical analyses were carried out using R 3.5.1. Data manipulation was achieved using "tidyverse" and "dplyr" R packages. Graphs were created using R packages "ggplot2", statistics were plotted with "ggpubr" and the graphics were modified using "gghx4" and "ggtext". All enrichment tests were achieved using "clusterProfiler". Jaccard distances were calculated using the "vegan" R package. The network was created using "igraph", permuted with "BiRewire", and visualized using Cytoscape. Chi-square, Fisher's exact, and Wilcoxon rank-sum tests, as well as linear models and correlations, were calculated using the "stats" R package. The scripts to execute the main analyses conducted in this study are available on GitHub.

For manuscripts utilizing custom algorithms or software that are central to the research but not yet described in published literature, software must be made available to editors and reviewers. We strongly encourage code deposition in a community repository (e.g. GitHub). See the Nature Portfolio guidelines for submitting code & software for further information.

Data

Policy information about availability of data

All manuscripts must include a data availability statement. This statement should provide the following information, where applicable:

- Accession codes, unique identifiers, or web links for publicly available datasets
- A description of any restrictions on data availability
- For clinical datasets or third party data, please ensure that the statement adheres to our policy

Metagenomic paired-reads of 3,588 samples were downloaded from the National Center for Biotechnology Information's (NCBI) Sequence Read Archive (SRA) from a total of 26 Bioprojects: PRJEB17784, PRJNA339012, PRJNA356102, PRJEB18755, PRJNA196801, PRJNA290729, PRJNA690543, PRJEB12947, PRJEB7774, PRJEB7949, PRJEB10878, PRJNA328899, PRJNA321058, PRJEB15371, PRJNA305507, PRJEB2054, PRJEB1786, PRJEB12124, PRJNA319574, PRJNA422434, PRJNA278393, PRJEB4336, PRJEB1220, PRJNA324129, PRJNA299502, PRJNA361402.
The relevant metadata, plasmid sequence files, and in-house plasmid gene database, are all available on GitHub. Plasmid annotations were achieved using the Kyoto Encyclopedia of Genes and Genomes (KEGG) KOfam database version 4. AMR gene predictions were based on the Comprehensive Antibiotic Resistance Database (CARD) v3.2.5.

Research involving human participants, their data, or biological material

Policy information about studies with human participants or human data. See also policy information about sex, gender (identity/presentation), and sexual orientation and race, ethnicity and racism.

Reporting on sex and gender	not applicable
Reporting on race, ethnicity, or other socially relevant groupings	not applicable
Population characteristics	not applicable
Recruitment	not applicable
Ethics oversight	not applicable

Note that full information on the approval of the study protocol must also be provided in the manuscript.

Field-specific reporting

Please select the one below that is the best fit for your research. If you are not sure, read the appropriate sections before making your selection.

[x] Life sciences   [ ] Behavioural & social sciences   [ ] Ecological, evolutionary & environmental sciences

For a reference copy of the document with all sections, see nature.com/documents/nr-reporting-summary-flat.pdf

Life sciences study design

All studies must disclose on these points even when the disclosure is negative.

Sample size	Metagenomic paired-reads of 3,588 samples were downloaded from the National Center for Biotechnology Information's (NCBI) Sequence Read Archive (SRA) from a total of 26 Bioprojects, spanning different continents and diseases associated with dysbiosis. This breadth of data ensured a comprehensive representation of various microbiome compositions and disease states, making it suitable for the analysis.
Data exclusions	Samples with read depths below 2 million were discarded from the analyses, as shallow depths may not provide sufficient coverage for accurate assembly of contigs and plasmids, compromising the reliability of downstream analyses. This exclusion resulted in 3,467 samples.
Replication	not applicable to this study, as the datasets were obtained based on predetermined disease states and participant numbers, precluding the need for these methodologies.
Randomization	not applicable to this study, as the datasets were obtained based on predetermined disease states and participant numbers, precluding the need for these methodologies.
Blinding	not applicable to this study, as the datasets were obtained based on predetermined disease states and participant numbers, precluding the need for these methodologies.

Reporting for specific materials, systems and methods

We require information from authors about some types of materials, experimental systems and methods used in many studies. Here, indicate whether each material, system or method listed is relevant to your study. If you are not sure if a list item applies to your research, read the appropriate section before selecting a response.

Materials & experimental systems

n/a	Involved in the study	
☒	☐	Antibodies
☒	☐	Eukaryotic cell lines
☒	☐	Palaeontology and archaeology
☒	☐	Animals and other organisms
☒	☐	Clinical data
☒	☐	Dual use research of concern
☒	☐	Plants

Methods

n/a	Involved in the study	
☒	☐	ChIP-seq
☒	☐	Flow cytometry
☒	☐	MRI-based neuroimaging
